# Perception of Women regarding Respectful Maternity Care during Facility-Based Childbirth

**DOI:** 10.1155/2020/5142398

**Published:** 2020-07-04

**Authors:** Pratima Pathak, Bijaya Ghimire

**Affiliations:** Nepal Medical College, Department of Nursing, Kathmandu University, P. O. Box 13344, Fax No. 977-1-4912118, Jorpati, Kathmandu, Nepal

## Abstract

**Background:**

Respectful care during childbirth has been described as “a universal human right that encompasses the principles of ethics and respect for women's feelings, dignity, choices, and preferences.” Many women experience a lack of respectful and abusive care during childbirth across the globe.

**Objective:**

This study aimed to determine women's perception of respectful maternity care (RMC) during facility-based childbirth.

**Method:**

A descriptive cross-sectional study was conducted among 150 mothers admitted to the maternity ward of Nepal Medical College and Teaching Hospital using a purposive sampling technique. Data were collected through an interview technique by using a validated tool containing 15 items each measured on a scale of 5. Statistical Package for Social Science (SPSS) version 16 was used for data analysis. Frequency, percentage, mean score, standard deviation, chi-square test, and binary logistic regression were used to analyze descriptive and inferential statistics.

**Results:**

In total, 84.7% of the women reported that they have experienced overall RMC services with a mean score ± SD (61.70 ± 12.12). Though the majority of the women reported that they have experienced RMC services, they also revealed that they have experienced disrespectful care in various forms such as being shouted upon (30.0%), being slapped (18.7%), delayed service provision (22.7%), and not talking positively about pain and relief during childbirth (28.0%). Likewise, length of stay, parity, and time of delivery were found as factors that influenced friendly care (COR = 0.383, 95% CI: 0.157–0.934), abuse-free care (COR = 3.663, 95% CI: 1.607–8.349), and timely care (COR = 2.050, 95% CI: 1.031–4.076) dimensions of RMC, respectively.

**Conclusion:**

Even though RMC emphasizes eliminating disrespectful and abusive environment from health facilities, 15.0% of participants perceived that they have not experienced overall RMC services. So, the health facility should focus on the interventions which ensure that every woman receives this basic human dignity during one of the most vulnerable times in their lives.

## 1. Introduction

About 830 deaths of women are recorded around the globe every day, which is resulted by hurdles associated with pregnancy and childbirth. Ninety nine percentage of the total deaths are recorded to be occurring in developing countries [1]. Although the countries have been successful in decreasing the maternal mortality by less than 44.0% over for 25 years (1990–2015), they have integrated to drop down the global maternal mortality ratio to less than 70 per 100,000 live births in the year from 2016 to 2030 as a part of Sustainable Development Goals (SDG) [2]. In 2016, the maternity mortality ratio (MMR) in Nepal was 239 per 100,000 live births [3]. Though the percentage of deliveries conducted in health institutions was increased from 35 in 2011 [4] to 55 in 2016 [3], Nepal is focused on attaining 70 percent of all deliveries by SBAs and at organizations by 2020 to accomplish the SDG target [3].

Health institutions face several challenges with an increased number of women delivering in a health facility. It necessitates a greater effort to upgrade the level of care provided to mothers along with their rights to noble and dutiful care [5]. Women's choice of picking the health facility for labor had the highest influence on respectful health workers' behavior [6]. Meagre source has indicated health care providers' attitude, impoliteness, lack of confidentiality, differentiation based on cultural and religious grounds, physical abuse, dirty facilities, and retarded and slow care service provision to be the reasons for not choosing health facilities during labor [7, 8].

After the extensive research that indicates adequate proof on women mistreatment during pregnancy and childbirth, the World Health Organization (WHO) published a statement on stoppage and eradication of such disrespect and abuse (D&A) occurring in health institutes [5]. The statement emphasizes the government and development partners for the initiation of support and sustains programs to deliver quality maternal and newborn health services. The statement also highlights the provision of respectful maternity care (RMC) as a significant element of the quality of care [5].

Humble and dutiful care during childbirth has been termed as “a universal human right that encompasses the principles of ethics and respect for women's feelings, dignity, choices, and preferences [9–11].” RMC is centered on increasing the interpersonal interactions between a woman and health care providers during various stages of childbirth such as labor, delivery, and postpartum. RMC predominantly focuses on the elimination of ill and abusive behavior by health care providers and associated staffs along with a sensitive and encouraging working environment to make a woman feel satisfied during her childbirth experience [12].

The range of disrespect and abuse encountered by women during childbirth in health institutions is well defined and classified. It consists of physical abuse (beating, slapping, and pinching), undignified care (yelling, chiding, and humiliating comments), abandonment (leaving alone during different phases of delivery), and discrimination based on the cultural group, social status, age, or restriction of facilities for nonfulfillment of fees [13, 14].

In Nepal, an estimate of the pervasiveness of respectful and nonabusive behavior during childbirth in health facilities has not been retrieved yet. This is, therefore, a significant topic to research upon, to know the forms of disrespect and abuse that prevail in the country. Identification of such behaviors will enlighten the respective personnel to formulate rules and regulations for the eradication of such ill manners in health premises. In the long run, it will help to enhance the quality of maternity care and encourage women for facility-based childbirth.

## 2. Methods

### 2.1. Study Design, Setting, and Sample Size

A descriptive cross-sectional study was done in Nepal Medical College and Teaching Hospital (NMCTH), Kathmandu, to identify the level of perception of women admitted in the maternity ward regarding respectful maternity care during the childbearing period. NMCTH is situated at Attarkhel, Jorpati, about 11 km northeast of the Kathmandu city. NMCTH is a tertiary-level hospital implementing a safe motherhood programme under the government of Nepal. A total of 3,723 childbirths took place in the year 2019 AD as per the hospital delivery record register. This hospital serves as the referral center for emergency obstetric care services. The sample size was estimated by using the following formula for definite proportion:

(1) $$\begin{array}{c} n=\frac{Z^{2}pq}{d^{2}}, \end{array}$$

with the assumptions of 6% standard allowable error, 95% confidence, and 10% non response rate. The estimated prevalence of disrespect and abuse that a mother can face during childbirth is taken as 15%. This figure was taken from the cross-sectional study conducted in one of the large referral hospitals of Tanzania [15]. The required sample size was 150 by putting the values in the above formula.

### 2.2. Study Participants and Sampling Procedure

Purposive sampling technique was used to select the study participants in this study. Study participants were women admitted to the maternity ward who had a vaginal delivery and were about to discharge from the study facility. Women who had a delivery of their child via elective or emergency caesarean section or any other extreme complication that necessitated transfer to the operation theatre were excluded from this study to maintain similarity between the services provided to the study participants. The participants were screened for their eligibility to participate in the study. This included reviewing the participant's information from their medical records like the mode of delivery, an obstetric complication that necessitates transfer to the operation theatre, and instruction for discharge. All the women who met the inclusion criteria were recruited in the study by the researchers.

### 2.3. Data Collection

Face-to-face interview technique was used for data collection from all the mothers. The data were collected from November 17, 2018, to March 12, 2019. The researchers introduced themselves to the participants, explained the objectives of the study clearly, and obtained verbal informed consent from each participant before data collection. Data were collected immediately before discharge from the health facilities after childbirth to prevent recall bias. A validated tool containing 15 items each measured on a scale of 5 was used to measure women's perceptions regarding RMC. The scale had four dimensions: friendly care, abuse-free care, timely care, and discrimination-free care each consisting of a total of 7, 3, 3, and 2 items, respectively, and thus a total of 15 items [16].

To obtain a score for each dimension and overall perceptions of RMC, the raw score was transformed into a scale from 0 (lower) to 100 (higher) where1 = 0, 2 = 25, 3 = 50, 4 = 75, and 5 = 100. The perception of women for each component and overall perceptions towards RMC were determined in the standard manner by using the formula for transformation. Likewise, to find the level of perception of women regarding RMC, those women who scored 50 or more transformed score were categorized as “experienced RMC” and those women who scored less than 50 were categorized as “not experienced RMC.” Women's perception regarding RMC during facility-based childbirth in this study was operationally defined as the opinion of women regarding the respectful care they experienced during facility-based childbirth from their perspective. RMC measuring scale which was available only on the English version was translated in the Nepali version. It was done by consulting with Nepali language experts and subject experts for language verification.

Although the RMC scale is a validated tool, further validation of the tool in the local context was done by reviewing the literature and consulting with subject professionals. Also, pretesting was conducted in 10% of the total sample size, i.e., among 15 mothers in the maternity ward of NMCTH, immediately prior to discharge from the health facilities after childbirth, and those respondents who took part in pretest were excluded from the real study. Based on the pretesting, practicability and usability of the instrument were customized as needed. Cronbach's alpha test was used to maintain the internal consistency reliability of the tool which was found to be 0.75.

### 2.4. Data Analysis

Data were entered in Epi data 3.1, and entered data were exported to IBM SPSS version 16. Descriptive and inferential statistics were used for statistical analysis. Descriptive statistics such as frequency, percentage, and mean score were applied to find out sociodemographic and obstetric-related information and perception of women regarding respectful maternity care during childbirth. Inferential statistics such as the chi-square test and binary logistic regression were applied to find the association between perception of women regarding respectful maternity care during childbirth with selected sociodemographic and obstetric-related information. A *p* value of less than 0.05 was regarded as the appropriate level of statistical significance, and the strength of statistical association was assessed by odds ratios with 95% confidence intervals.

### 2.5. Ethical Consideration

Ethical clearance and formal approval for conducting research were obtained from the “Institutional Review Committee” of the Nepal Medical College. Approval letter for data collection was also obtained from the concerned authority of the organization from where those data were collected. Verbal informed consent was taken from each participant, and assurance of confidentiality and anonymity was ensured before data collection. Permission to use the data collection tool was obtained from the author.

## 3. Results

Table 1 shows the sociodemographic characteristics of the respondents. Most of the respondents, i.e., 41.4%, were of the age group 20 to 24 years. The average age of participants was 25.44, and the standard deviation was 4.91. Similarly, 61.3% of participants were from Janjati ethnicity, followed by Brahmin/Chhetri and then Dalit. In the same way, 64.7% of the participants were Hindus, and 35.3% of them were Buddhists. Likewise, 10.7% of the mothers were uneducated, while 54.0% of them claimed to have had education up to the secondary level. Sixty three point three percent of the participants stated household work as their occupation. In response to monthly family income, 78.0% of them responded to the income range between Rs. 10,000 and 40,000. Mean and SD of income was calculated to be Rs. 34,400.00 and 20276.61, respectively.

Table 2 presents the obstetric characteristics of the respondents. Most of the respondents (98.0%) had visited a health facility for their recent childbirth. Among the respondents who had their ANC visits in a health facility, 90.5% had more than four ANC visits. Likewise, 61.3% of the respondents had stayed for longer than one day in a health facility for their recent delivery, and 38.0% of the respondents had their delivery during the night shift. Also, only 58 mothers were reported to have had childbirth experience previously. Among the mothers who had given birth previously, 72.4% had 1 to 2 no. of living children. Likewise, 82.8% of mothers reported having had their delivery in health facilities during their previous childbirth.

Table 3 shows the mean score and SD of each dimension, as well as the overall dimensions of RMC. A majority, i.e., 84.7%, of the women stated that they had perceived overall dimensions of RMC with the mean score being 61.70 with a standard deviation of 12.12. Likewise, among the four dimensions of RMC, the highest average score of 69.00 is observed in the discrimination-free care dimension with the SD of 15.84, where 80.7% of the women claimed being received discrimination-free care.

Table 4 illustrates the perception of mothers in each item of four dimensions of RMC. In the friendly care dimension, 83.3% of the participants agreed that health workers spoke to them in a language they could understand. Under dimension 2, i.e., abuse-free care, 54.0% of the participants agreed that health care professionals acknowledged their needs irrespective of their request. In the same way, among the three statements used under the timely care dimension of RMC, 68.0% of the mothers answered that they do not know if they can practice their cultural rituals in the health facility. Under another component of RMC, discrimination-free care, 76.7% of women strongly disagreed that they were poorly treated based on their personal attributes.

This study tested the relationship between the level of perception on overall RMC and each dimension of RMC with different variables such as age, ethnicity, education, occupation, monthly family income, length of stay during their recent childbirth, time of delivery, and parity through the chi-square test of independence and logistic regression analysis. However, the result does not show the statistical association between overall RMC and different variables and also between the discrimination-free care component of RMC and with variables being studied. Only the length of stay of the respondents for their recent childbirth in the hospital was found to be statistically significant with the friendly care (*p*=0.031) component of RMC. The result of binary logistic regression analysis also showed that respondents who stayed for the shorter time period in the hospital that is one or less than one day were more likely to experience the friendly care component of RMC than those who stayed for longer than one day (COR = 0.383, 95% CI: 0.157–0.934) (Table 5).

Table 6 presents the results of the association of maternal characteristics with the abuse-free care components of RMC. The data depicts that having living children previously with the respondents is significantly associated with the abuse-free component of RMC. Similarly, the results of binary logistic regression analysis also showed that mothers who have at least one or more children are 3.663 times more likely to experience abuse-free care component of RMC than those mothers who have not given birth to a child previously (COR = 3.663, 95% CI: 1.607–8.349).

Table 7 represents the results of the association of different variables with timely care components of RMC. However, the results showed that there is a statistically significant association between only the time of delivery and the timely care component of RMC among the several variables (*p*=0.039). Furthermore, the results of binary logistic regression analysis also showed that respondents giving birth to the baby in the day shift were 2.050 times more likely to experience timely care components of RMC than those giving birth in the night shift (COR = 2.050, 95% CI: 1.031–4.076).

## 4. Discussion

This study intended to measure the level of perception of women on RMC during childbirth. Over three-quarters of women interviewed during the study reported to have experienced overall dimensions of RMC during their recent childbirth in this study. Their perceptions were measured on the four main dimensions of RMC, i.e., friendly care, abuse-free care, timely care, and discrimination-free care. Article IV of the UN's universal rights of childbearing women document states that every woman has the right to be treated with dignity and respect [9]. However, in this study, still 15.0% of women concurred that they have not experienced the overall dimensions of RMC.

The present study revealed that women received various forms of nonfriendly care during childbirth, ranging from not showing concern and empathy (24.7%) to not talking positively about pain and the relief (28.0%) to the childbearing women. A similar pattern of nonrespectful care has been reported in Addis Ababa, Ethiopia [17]. These kinds of nonrespectful care reported in the study might have negative consequences for service utilization [14]. Moreover, there is documented evidence that supportive behavior during childbirth positively influences birth outcomes. Hence, disrespectful behaviors affect birth outcomes negatively [18, 19]. Likewise, only 4.0% of women in this study said that health workers spoke to them in a nonunderstandable language, and this proportion is lower than that in South Africa [20] and Addis Ababa, Ethiopia [17].

Physical and verbal abuse, which is often neglected, has unacceptable and harmful impacts and is likely to contribute to the higher rates of unnecessary interventions and traumatic birth experiences [21]. In the study carried out, 18.7% of women strongly agreed that they were slapped during childbirth. This proportion is in line with the outcome of a similar study carried out in Addis Ababa, Ethiopia, where 23.0% of participants reported that health workers used physical force (slap/hit) [17]. The finding of this study is much higher than the observational study conducted in Tanzania [22] and Ethiopia [23]. The lesser percentage in Tanzania and Ethiopia can be justified by observational effects. Had there been no observers, the percentage could have been higher. Nevertheless, it can only be confirmed when further research which could avert observers' effect is carried out.

Similarly, 30.0% of women strongly agreed on being shouted by health care providers in this study. This result is slightly higher than that of Kenya where only 18.0% of women were recorded to be verbally abused (shouting) [24]. The finding of this study is much higher than that of verbal abuse rates reported in a similar study in Ethiopia where only 8.0% of women were recorded to have experienced such abuse [23]. The higher rate of verbal abuse in the study is unexpected, and it needs further investigation as to why health workers are committing such actions.

Continued support of the health care providers during childbirth has shown clinically meaningful benefits for the health of a woman and her newborn as per the findings of the systematic review on the importance of continuous support during childbirth [25]. Despite its critical benefits, a considerable proportion, i.e., 22.7%, of women in this study strongly agreed on their inattention by health workers. A similar figure was observed in studies carried out in south-eastern Nigeria [26] and four health facilities of Addis Ababa, Ethiopia [17], where 29.1% and 23.1% of women, respectively, were recorded to have been left alone or unattended by the care provider. This result is quite different from similar studies conducted in Kenya [24] and Tanzania [15]. Mothers unattended by health workers in this study might be a result of staff constraints or overcrowding rather than intentional. Short staffs diminish staff efficiency and eventually deteriorate the timely service to mothers. Furthermore, these types of constraints might create stressful working conditions which may predispose the health care providers to behave poorly with women [25].

There are a lot of examples that show how discrimination based on one's race, ethnicity, religion, age, socioeconomic status, and HIV status is still prevalent in the health facility [14]. In this study, 13.3% of the women strongly cited they were not treated fairly because of their personal attribute which supports the study conducted in Nigeria where the percentage was 20.0% [26]. Mothers might prefer to deliver at their residence to avoid embarrassment and discrimination in health facilities. Many studies have also concluded the fear of such discrimination to be a key barrier to facility-based deliveries in low- and middle-income countries [27]. Maternity care experts and program managers should highlight the diversity and promote equity for every defenceless groups, with continuous observation and assessment of respectful care in units.

This study tested the relationship between age, ethnicity, religion, education, monthly family income, occupation, parity, length of stay for delivery, and time of delivery with the prevalence of different dimensions of the RMC and the overall prevalence of the RMC. There are no statistical associations between different dimensions of the RMC with women's age, ethnicity, religion, education, monthly family income, and occupation. However, in this study, length of stay during delivery, time of delivery, and parity were found to be statistically significant with the dimensions of the RMC.

The study illustrates that the women who stayed at the facility for one or less than one day were more likely to experience friendly care than those who stayed longer (COR = 0.383, 95% CI: 0.157–0.934). This finding of the study is similar to that of the study conducted in Tanzania [28]. In addition to this, mothers who have already given birth to a child previously are 3.663 times more likely to receive abuse-free care than those of new mothers (COR = 3.663, 95% CI: 1.607–8.349). This discrepancy might be because mothers who have gone through the childbirth process previously are more likely to understand and obey health professionals quickly, and thus less likely to receive abusive care. Also, mothers who have already given birth to a child are more likely to have easier and quicker deliveries compared to new mothers. This demonstrates the complexities of the childbirth process along with the necessities of friendly and abuse-free care from the health care personnel who might have overlooked it and were impolite. Nonetheless, the result is in contrast with a similar study led in Kenya where mothers with higher parity were more likely to experience disrespect and abuse than new mothers [24].

The study also reveals mothers giving birth to the baby at day time experienced timely care than those giving birth at night time (COR = 2.050, 95% CI: 1.031–4.076). This result is in line with the result from a study conducted in Kenya [24]. The reason might be because during the night time, staffs are more likely to have work overload as staffs are generally lower during the night shift in a low-resource country like Nepal. Lesser management supervision during the night time may predispose to some extent for such laxity in timely care.

The primary strength of this study is the reduced possibility of recall bias as women were interviewed immediately before discharge after their childbirth. Only one study setting was included in this study which might influence the result of this study and may limit its generalizability. Even though mothers participated in the study after they were assured that their personal information will be kept confidential and their opinions will solely be for study purposes, the responses might have been influenced by courtesy bias or the unwillingness of women to report any negative experiences while still at the facility. Some of the important dimensions of RMC identified by the literature review (consented care and confidential care) could not be identified in the study as the standard tool was being used in the study.

## 5. Conclusion

The findings of the study showed that over three-quarters of the women reported that they have experienced overall respectful maternity care services. Even though the majority of the women experienced the overall dimensions of RMC, verbal abuse, physical abuse, delayed service provision, and not talking positively about pain and relief were some of the aspects of disrespectful care reported being experienced by women in the study. Likewise, length of stay for delivery, time of delivery, and parity were identified as factors that influenced friendly care, timely care, and abuse-free care dimensions of RMC, respectively. Understanding the prevalence and status of RMC services is very crucial in developing interventions in different levels of the health facility and to encourage clients' for future use of the health facility during the childbearing time. It is every woman's right to give birth in a context free from disrespect and abuse. Hence, the provision of woman's centered care in a respectful and nonabusive manner needs to be given adequate emphasis to make service more qualitative and woman-friendly.

## Figures and Tables

**Table 1:** Sociodemographic characteristics of the respondents *n* = 150.

Characteristics	Number	Percentage
Age in years		
15–19	15	10.0
20–24	62	41.4
25–29	44	29.3
30–34	20	13.3
35–39	9	6.0
Mean age = 25.44		
SD = ±4.91		
Ethnicity		
Brahmin/Chhetri	48	32.0
Janjati	92	61.3
Dalit	10	6.7
Religion		
Hinduism	97	64.7
Buddhism	53	35.3
Educational status		
No formal education	16	10.7
Up to secondary level	81	54.0
Higher secondary and above	53	35.3
Occupation		
Household work	95	63.3
Business	25	16.7
Service	14	9.4
Agriculture	11	7.3
Others	5	3.3
Monthly family income (Rs.)		
10,000 to 40,000	117	78.0
40,000 to 70,000	25	16.7
70,000 to 100,000	8	5.3
Mean income = 34,400.00		
SD = ±20276.61		

**Table 2:** Obstetric characteristics of the respondents *n* = 150.

Characteristics	Number	Percentage
Status of ANC visit for recent childbirth		
Visited health facility for ANC	147	98.0
Not visited the health facility for ANC	3	2.0

No. of ANC visit (*n* = 147)		
Up to 4	14	9.5
More than 4	133	90.5

Length of stay for the recent delivery		
≤1 day	58	38.7
>1 day	92	61.3

Time of delivery		
Morning	49	32.7
Evening	44	29.3
Night	57	38.0

Previous parity (*n* = 58)		
1-2	42	72.4
2–4	16	27.6

Place of delivery of the previous child (*n* = 58)		
Health facility	48	82.8
Home	10	17.2

**Table 3:** Level of perception on overall and four dimensions of RMC *n* = 150.

Variables	Experienced RMC *n* (%)	Not experienced RMC *n* (%)	Mean ± SD
Friendly care	126 (84.0)	24 (16.0)	64.83 ± 15.53
Timely care	97 (64.7)	53 (35.3)	56.55 ± 17.09
Abuse-free care	104 (69.3)	46 (30.7)	56.44 ± 19.64
Discrimination-free care	121 (80.7)	29 (19.3)	69.00 ± 15.84
Overall RMC	127 (84.7)	23 (15.3)	61.70 ± 12.12

**Table 4:** Perception regarding respectful maternity care among respondents *n* = 150.

RMC item	SD (%)	D (%)	DK (%)	A (%)	SA (%)
Dimension 1: friendly care					
Cared with a kind approach	2 (1.3)	11 (7.3)	14 (9.3)	107 (71.3)	16 (10.8)
Treated in a friendly manner	3 (2.0)	25 (16.7)	19 (12.7)	92 (61.3)	11 (7.3)
Talked positively about pain and relief	3 (2.0)	42 (28.0)	15 (10.0)	81 (54.0)	9 (6.0)
Showed concern and empathy	4 (2.7)	36 (24.0)	13 (8.6)	91 (60.7)	6 (4.0)
Treated me with respect as an individual	3 (2.0)	37 (24.7)	14 (9.3)	88 (58.7)	8 (5.3)
Spoke to me in a language that I could understand	0 (0.0)	6 (4.0)	2 (1.4)	125 (83.3)	17 (11.3)
Called me by my name	6 (4.0)	19 (12.7)	15 (10.0)	97 (64.7)	13 (8.6)

Dimension 2: abuse-free care					
Responded to my needs whether or not I asked	6 (4.0)	36 (24.0)	20 (13.3)	81 (54.0)	7 (4.7)
Slapped me (R)	105 (70.0)	5 (3.3)	5 (3.3)	7 (4.7)	28 (18.7)
Shouted at me (R)	77 (51.3)	3 (2.0)	4 (2.7)	21 (14.0)	45 (30.0)

Dimension 3: timely care					
Kept waiting for a long time before getting service (R)	91 (60.7)	4 (2.7)	5 (3.3)	14 (9.3)	36 (24.0)
Allowed to practice cultural rituals in the facility	0 (0.0)	3 (2.0)	102 (68.0)	43 (28.7)	2 (1.3)
Service provision was delayed (R)	93 (62.0)	1 (0.7)	11 (7.3)	11 (7.3)	34 (22.7)

Dimension 4: discrimination-free care					
Not treated me well because of some personal attribute (R)	115 (76.7)	6 (4.0)	9 (6.0)	0 (0.0)	20 (13.3)
Insulted me and my companions because of my personal attribute (R)	117 (78.0)	8 (5.3)	13 (8.7)	0 (0.0)	12 (8.0)

SD = strongly disagree, D = disagree, DK = do not know, A = agree, SA = strongly agree, and (R): the item is reverse coded.

**Table 5:** Association of maternal characteristics with the friendly care dimension of RMC *n* = 150.

Variables	Experienced RMC *n* (%)	Not experienced RMC *n* (%)	*χ* ^2^ (*p* value)	COR (95% CI)
Age				
≤25	71 (83.5)	14 (16.5)	0.032 (0.857)	Ref
>25	55 (84.6)	10 (15.4)		1.085 (0.448–2.627)

Educational status				
Up to secondary level	83 (85.6)	14 (14.4)	0.502 (0.479)	Ref
More than secondary level	43 (81.1)	10 (18.9)		0.725 (0.298–1.768)

Parity				
No previous children	76 (82.6)	16 (17.4)	0.343 (0.558)	Ref
Between 1 and 4 children	50 (86.2)	8 (13.8)		1.316 (0.524–3.304)

Time of delivery				
Day shift	79 (84.9)	14 (15.1)	0.163 (0.686)	1.201 (0.494–2.918)
Night shift	47 (82.5)	10 (17.5)		Ref

Length of stay				
≤1 day	44 (75.9)	14 (24.1)	4.660 (0.031^*∗*^)	0.383 (0.157–0.934)^*∗∗*^
>1 day	82 (89.1)	10 (10.9)		Ref

^*∗*^ *p* value is significant at ≤0.05 level, Ref: reference group, ^*∗∗*^significant at 95% CI, and COR = crude odds ratio.

**Table 6:** Association of maternal characteristics with abuse-free care dimension of RMC *n* = 150.

Variables	Experienced RMC *n* (%)	Not experienced RMC *n* (%)	*χ* ^2^ (*p* value)	COR (95% CI)
Age				
≤25	58 (68.2)	27 (31.8)	0.111 (0.739)	Ref
>25	46 (70.8)	19 (29.2)		1.127 (0.558–2.227)

Educational status				
Up to secondary level	66 (68.0)	31 (32.0)	0.216 (0.642)	Ref
More than secondary level	38 (71.7)	15 (28.3)		1.190 (0.571–2.480)

Parity				
No previous children	55 (59.8)	37 (40.2)	10.207 (0.001^*∗*^)	Ref
Between 1 and 4 children	49 (84.5)	9 (15.5)		3.663 (1.607–8.349)^*∗∗*^

Time of delivery				
Day shift	65 (69.9)	28 (30.1)	0.036 (0.850)	1.071 (0.525–2.186)
Night shift	39 (68.4)	18 (31.6)		Ref

Length of stay				
≤1 day	38 (65.5)	20 (34.5)	0.648 (0.421)	1.336 (0.659–2.708)
>1 day	66 (71.7)	26 (28.3)		Ref

^*∗*^ *p* value is significant at ≤0.05 level, Ref: reference group, ^*∗∗*^significant at 95% CI, and COR = crude odds ratio.

**Table 7:** Association of maternal characteristics with timely care dimension of RMC *n* = 150.

Variables	Experienced RMC *n* (%)	Not experienced RMC *n* (%)	*χ* ^2^ (*p* value)	COR (95% CI)
Age				
≤25	53 (62.4)	32 (37.6)	0.460 (0.498)	Ref
>25	44 (67.7)	21 (32.3)		1.265 (0.641–2.498)

Educational status				
Up to secondary level	63 (64.9)	34 (35.1)	0.010 (0.922)	Ref
More than secondary level	34 (64.2)	19 (35.8)		0.966 (0.480–1.944)

Parity				
No previous children	57 (62.0)	35 (38.0)	0.765 (0.382)	Ref
Between 1 and 4 children	40 (69.0)	18 (31.0)		1.365 (0.679–2.741)

Time of delivery				
Day shift	66 (71.0)	27 (29.0)	4.253 (0.039^*∗*^)	2.050 (1.031–4.076)^*∗∗*^
Night shift	31 (54.4)	26 (45.6)		Ref

Length of stay				
≤1 day	36 (62.1)	22 (37.9)	0.279 (0.597)	1.203 (0.607–2.384)
>1 day	61 (66.3)	31 (33.7)		Ref

^*∗*^ *p* value is significant at ≤0.05 level, Ref: reference group, ^*∗∗*^significant at 95% CI, and COR = crude odds ratio.

## Data Availability

The data used to support the findings of this study are available from the corresponding author upon request.

## References

[1] Say L., Chou D., Gemmill A. (2014). Global causes of maternal death: a WHO systematic analysis. *The Lancet Global Health*.

[2] World Health Organization (2016). *Maternal Mortality: Fact Sheet 2016*.

[3] Government of Nepal (2015). Annual health report department of health services 2072/73 (2015/2016).

[4] Ministry of Health and Population (2011). *Nepal Demographic and Health Survey 2011*.

[5] World Health Organization (2015). *The Prevention and Elimination of Disrespect and Abuse during Facility Based Childbirth 2015*.

[6] Kruk M. E., Paczkowski M., Mbaruku G., Pinho H. D., Galea S. (2009). Women’s preferences for place of delivery in rural Tanzania: a population-based discrete choice experiment. *American Journal of Public Health*.

[7] Gebrehiwot T., Goicolea I., Edin K., Sebastian M. S. (2012). Making pragmatic choices: women’s experiences of delivery care in Northern Ethiopia. *BMC Pregnancy Childbirth*.

[8] Mselle L. T., Moland K. M., Mvungi A., Evjen-Olsen B., Kohi T. W. (2013). Why give birth in health facility? Users’ and providers’ accounts of poor quality of birth care in Tanzania. *BMC Health Services Research*.

[9] White Ribbon Alliance (2011). *Respectful Maternity Care: The Universal Rights of Childbearing Women*.

[10] Windau-Melmer T. (2013). *A Guide for Advocating for Respectful Maternity Care*.

[11] Reis V., Deller B., Carr C., Smith J. (2012). *Respectful Maternity Care: Country Experiences: Survey Report*.

[12] White Ribbon Alliance (2015). *Pulling Back the Curtain on Disrespect and Abuse: The Movement to Ensure Respectful Maternity Care*.

[13] Bohren M. A., Vogel J. P., Hunter E. C. (2015). The mistreatment of women during childbirth in health facilities globally: a mixed-method systematic review. *PLOS Medicine*.

[14] Bowser D., Hill K. (2010). *USAID: Exploring Evidence for Disrespect and Abuse in Facility-Based Childbirth: Report of a Landscape Analysis*.

[15] Sando D., Ratcliffe H., Donald K. M. (2016). The prevalence of disrespect and abuse during facility-based childbirth in urban Tanzania. *BMC Pregnancy Childbirth*.

[16] Sheferaw E. D., Mengesha T. Z., Wase S. B. (2016). Development of a tool to measure women’s perception of respectful maternity care in public health facilities. *BMC Pregnancy Childbirth*.

[17] Asefa A., Bekele D. (2015). Status of respectful and non-abusive care during facility-based childbirth in a hospital and health centers in Addis Ababa, Ethiopia. *Reproductive Health*.

[18] Barrett S. J., Stark M. A. (2010). Factors associated with labor support behaviors of nurses. *Journal of Perinatal Education*.

[19] Sleutel M. R. (2002). Development and testing of the labor support scale. *Journal of Nursing Measurement*.

[20] Oosthuizen S. J., Bergh A.-M., Pattinson R. C., Grimbeek J. (2017). It does matter where you come from: mothers’ experiences of childbirth in midwife obstetric units, Tshwane, South Africa. *Reproductive Health*.

[21] Hodges S. (2009). Abuse in hospital-based birth settings?. *Journal of Perinatal Education*.

[22] Sando D., Kendall T., Lyatuu G. (2014). Disrespect and abuse during childbirth in Tanzania: are women living with HIV more vulnerable?. *Journal of Acquired Immune Deficiency Syndromes*.

[23] Sheferaw E. D., Bazant E., Gibson H. (2017). Respectful maternity care in Ethiopian public health facilities. *Reproductive Health*.

[24] Abuya T., Ndwigal C., Ritter J. (2015). The effect of a multi-component intervention on disrespect and abuse during childbirth in Kenya. *BMC Pregnancy Childbirth*.

[25] Hodnett E. D., Gates S., Hofmeyr G. J., Sakala C. (2014). Continuous support for women during childbirth. *Cochrane Database Systematic Reviews*.

[26] Okafor I. I., Ugwu E. O., Obi S. N. (2015). Disrespect and abuse during facility-based childbirth in a low-income country. *International Journal of Gynecology & Obstetrics*.

[27] Bohren M. A., Hunter E. C., Munthe-Kaas H. M., Souza J. P., Vogel J. P., Gülmezoglu A. M. (2014). Facilitators and barriers to facility-based delivery in low-and middle-income countries: a qualitative evidence synthesis. *Reproductive Health*.

[28] Kruk M. E., Kujawski S., Mbaruku G., Ramsey K., Moyo W., Freedman L. P. (2018). Disrespectful and abusive treatment during facility delivery in Tanzania: a facility and community survey. *Health Policy and Planning*.

